# Combination Therapy and Nanoparticulate Systems: Smart Approaches for the Effective Treatment of Breast Cancer

**DOI:** 10.3390/pharmaceutics12060524

**Published:** 2020-06-08

**Authors:** Shivaprasad Gadag, Shristi Sinha, Yogendra Nayak, Sanjay Garg, Usha Y. Nayak

**Affiliations:** 1 Department of Pharmaceutics, Manipal College of Pharmaceutical Sciences, Manipal Academy of Higher Education, Manipal 576104, India; shivaprasad.gadag@learner.manipal.edu (S.G.); shristi.sinha@learner.manipal.edu (S.S.); 2 Department of Pharmacology, Manipal College of Pharmaceutical Sciences, Manipal Academy of Higher Education, Manipal 576104, India; yogendra.nayak@manipal.edu; 3 UniSA: Clinical and Health Sciences, University of South Australia, Adelaide, SA 5000, Australia; sanjay.garg@unisa.edu.au

**Keywords:** combination therapy, PI3K/AKT/mTOR inhibitors, monoclonal antibodies, immunotherapy, antibody-drug conjugates, nanomedicine

## Abstract

Breast cancer has become one of the biggest concerns for oncologists in the past few decades because of its unpredictable etiopathology and nonavailability of personalized translational medicine. The number of women getting affected by breast cancer has increased dramatically, owing to lifestyle and environmental changes. Besides, the development of multidrug resistance has become a challenge in the therapeutic management of breast cancer. Studies reveal that the use of monotherapy is not effective in the management of breast cancer due to high toxicity and the development of resistance. Combination therapies, such as radiation therapy with adjuvant therapy, endocrine therapy with chemotherapy, and targeted therapy with immunotherapy, are found to be effective. Thus, multimodal and combination treatments, along with nanomedicine, have emerged as a promising strategy with minimum side effects and drug resistance. In this review, we emphasize the multimodal approaches and recent advancements in breast cancer treatment modalities, giving importance to the current data on clinical trials. The novel treatment approach by targeted therapy, according to type, such as luminal, HER2 positive, and triple-negative breast cancer, are discussed. Further, passive and active targeting technologies, including nanoparticles, bioconjugate systems, stimuli-responsive, and nucleic acid delivery systems, including siRNA and aptamer, are explained. The recent research exploring the role of nanomedicine in combination therapy and the possible use of artificial intelligence in breast cancer therapy is also discussed herein. The complexity and dynamism of disease changes require the constant upgrading of knowledge, and innovation is essential for future drug development for treating breast cancer.

## 1. Introduction

Breast cancer (BC) is one of the most common cancers prevalent across the globe and is one of the leading causes of mortality and morbidity in women [1]. The incidence of BC cases is expected to increase mainly due to aging and population growth. It is expected that by 2030, the incidence rate increase by 50% [2]. The challenges met during treatment are very vivid because of the heterogeneous property of disease [3]. The reason behind the prevalence of the disease is the substantial individual variation. The incidence of breast cancer are on the rise due to a change in lifestyle and availability of modern screening techniques. Despite the advances in screening techniques, mortality rates are higher, especially in developing countries, due to a lack of easy access to state-of-the-art diagnosis and treatment facilities. In addition, a speedy cancer progression and metastasis can be the reasons for the reduced survival rate.

BC is classified based on features, namely, histopathology, molecular subtypes, stage of cancer, grade, and based on DNA alteration. The treatment point of view classification based on molecular subtypes is the most accepted. Molecular subtyping of breast cancer explains its four types of nature, namely, luminal breast cancer (LBC) type A, LBC type B, human epidermal growth factor receptor 2 (HER2) positive breast cancer, and triple-negative breast cancer (TNBC) [4]. The LBC is further subdivided into type-A and type-B, which differ in their etiology. Type-A shows a positive test only for hormone receptors (HR), which includes both progesterone and estrogen receptors. In the case of type-B, both HR and HER tests are positive. In addition to these two types, BC also shows a high amount of Ki-67 expression. LBC type-A progresses slowly and can be treated in its early stages. In contrast, type-B is highly progressive, and its early-stage detection is not usually possible, making it more fatal [5]. The third molecular subtype, HER2-positive cancer, has a predominant expression of HER2/ERBB2 oncogene. TNBC, the fourth molecular subtype, lacks both HR and HER2 and is considered to be the most aggressive form of BC. TNBC can further classified into six subclasses, namely, luminal androgen receptor subtype, immunomodulator, mesenchymal, stem-like mesenchymal, and basal-like (BL1 and BL2). According to cancer statistics, 84% of BCs are HR-positive cancers. Among the HR-positive diseases, 71% are affected by type-A LBC, and the rest, 12%, are affected by type-B LBC. Around 5% of the cancer patients are affected by HER2-positive and HR-negative, and the remaining 12% suffer from TNBC [6].

Based on various factors such as cancer stage, presence/absence of hormone receptors, genetic and epigenetic alterations, and biomarkers, different treatment strategies are adopted. Such strategies include surgery, chemotherapy, radiation therapy, hormone therapy, immunotherapy, targeted therapy used alone or in combination. An exhaustive set of literature is available on the treatment options for BC. In the first part of the present review, we discuss the current multimodal treatment options, including combination therapy for BC, followed by the newer strategies adopted. The lists of drugs that are under clinical development are detailed according to their clinical applicability. The recent research related to the utilization of nanotechnological platforms to deliver drugs effectively is included in the second part of this review.

## 2. Treatment Strategies for Breast Cancer

### 2.1. Current Treatment Approaches

#### 2.1.1. Therapeutic Options for Luminal Breast Cancer

Approximately 60% to 80% of premenopausal women with BC belong to the HR-positive type [5,6]. If breast cancer has a significant number of hormone receptors for estrogen or progesterone, it is classified as HR-positive. The growth of the tumor is fueled by estrogen and progesterone in HR-positive BC. The best-suited therapy for HR-positive BC is by targeting the estrogen receptors, and these hormone levels can either be reduced or blocked. The estrogen receptor (ER) blockers, such as tamoxifen, prevent the attachment of estrogen, arresting the BC growth [7]. Fulvestrant is another drug that belongs to the class of estrogen-degrading agents, which selectively destroy the estrogen receptors [8]. Another mechanism is by targeting the aromatase enzyme, which is responsible for the conversion of androgen to estrogen. The agents approved for this action are exemestane, anastrozole, and letrozole. Further, leuprolide and goserelin the potent gonadotropin-releasing hormone receptor (GnRHR) agonists have been approved, which inhibit hormone production from the ovary. However, the major limitation of endocrine therapy is the development of resistance, which causes the relapse of BC and poor clinical outcomes [9,10].

#### 2.1.2. Treatment Options for HER2-Positive BC

For the treatment of HER2-positive BC in its early stage, combinational therapy is the preferred treatment of choice, e.g., palbociclib in combination with hormone therapy (such as fulvestrant, an anti-estrogen drug, or letrozole, an aromatase inhibitor). However, the above treatment regimen is not enough for the complete removal of cancer cells. Following combinational therapy, surgery is performed, followed by radiotherapy and a year of continuous anti-HER2 targeted treatment. There are different drugs available for the treatment, namely, lapatinib, a tyrosine kinase receptor inhibitor that interrupts both HER2 and epidermal growth factor receptor (EGFR), and microtubule inhibitors linked with antibody cytotoxic agents, e.g., emtansine linked with the monoclonal antibody trastuzumab [4].

#### 2.1.3. Management of Triple-Negative Breast Cancer (TNBC)

TNBC is clinically and histopathologically different from the rest of BC, and it requires different treatment approaches. TNBC has the highest rates of recurrence, which makes it the most challenging type of BC to be treated [11]. Chemotherapy using taxanes, anthracycline, and platinum drugs remains the standard treatment for TNBC.. This conventional treatment can be combined with anti-VEGF monoclonal antibody bevacizumab. The vascular endothelial growth factor (VEGF) is oncogenic and can be a druggable target in BC [12]. Since TNBC lacks all the receptors, the use of targeted therapy to the receptor does not work. Thus, there is an inevitable need for newer, aggressive targeted technology for the better treatment of TNBC [4].

### 2.2. Novel Treatment Approaches for Breast Cancer by Targeted Therapy

**Figure 1:**
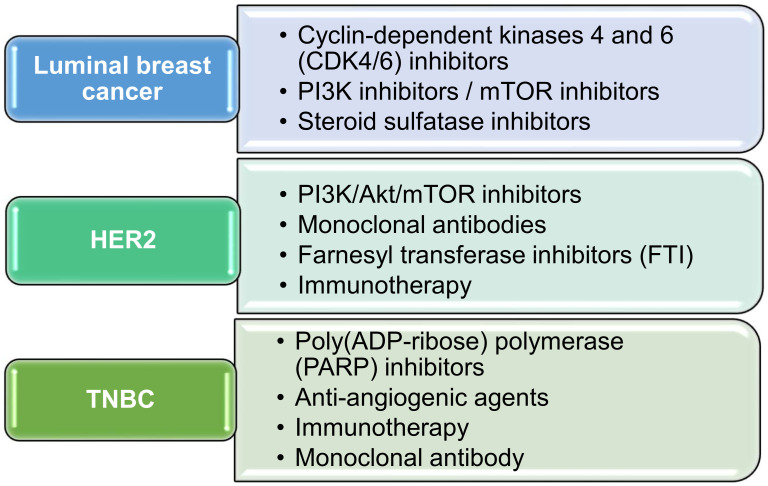
Classification of drugs under targeted therapy based on the type of breast cancer.

As the name indicates, targeted therapy refers to targeting specific biological macromolecules or biochemical pathways in cancer cell metabolism. Generally, there are two types of targeted therapy used: one is the small molecular targeted therapy, and the other is the use of monoclonal antibodies. The commonly used agents under targeted therapy for breast cancer are presented in Figure 1 and discussed in this section, according to the types of BC.

#### 2.2.1. Therapy of Luminal Breast Cancer (LBC)

##### CDK4/6 Inhibitors

**Figure 2:**
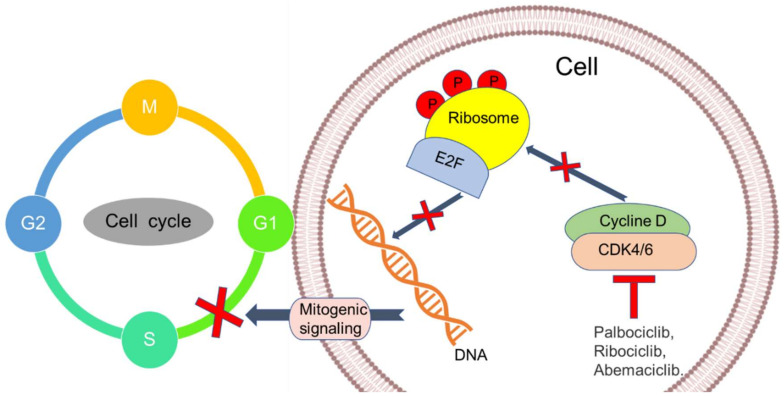
Schematic representation of the mechanism of CDK4/6 inhibitors. E2F—a group of genes that encodes a family of transcription factors, CDK4/6—cyclin-dependent kinase 4/6, DNA—deoxyribonucleic acid.

Cyclin-dependent kinase 4 and 6 (CDK4/6) inhibitors have become one of the prime targets for inhibiting cancer growth. It interacts reversibly with cyclin D1 and regulates cell cycle progression. When the tumor develops hormone resistance, they still depend on the proliferation of CDK4/6-cyclin D1 [13]. The first mechanism by which these inhibitors work is by the blocking of retinoblastoma (Rb) protein, which helps in the phosphorylation of kinases (Figure 2). The phosphorylation process leads to G1-S cell cycle arrest, which is very prominent in the treatment of BC as a combinational therapy, including CDK4/6 inhibitors and hormonal treatment. CDK4/6 also works by dephosphorylating Forkhead box protein M1, which further inhibits cell proliferation [14]. In phase III studies on metastatic breast cancer, the highly selective oral CDK4/6 inhibitors were examined in combination with endocrine therapy. The CDK4 (INK4)-Rb CDK4/6-inhibitor plays a vital role in the progression of the cell cycle, and its dysregulation is a significant contributor to the resistance of endocrine therapy. Palbociclib, ribociclib, and abemaciclib are the drugs that have been approved for the treatment of BC, which have shown a higher affinity and potency towards CDK4, as compared to CDK6 (Table 1) [15]. Clinical trials of these drugs revealed that the oral dosage showed a low and easily manageable toxicity [16]. Neutropenia and thrombocytopenia were the significant side effects in palbociclib- and ribociclib-treated patients while abemaciclib presented neutropenia, diarrhea, and fatigue as the major side effects. US FDA has approved ribociclib, abemaciclib, and palbociclib for HR-positive, HER2-negative, and metastatic BC (MBC) in July 2018, February 2018, and March 2017, respectively [17,18,19].

Combination therapy is a well-known option to reduce side effects and enhance therapeutic outcomes. Table 2 presents the clinical trials that are ongoing to ascertain the efficacy of combination therapy given along with CDK4/6 inhibitors.

##### PI3K/AKT/mTOR Pathway Inhibitors

**Figure 3:**
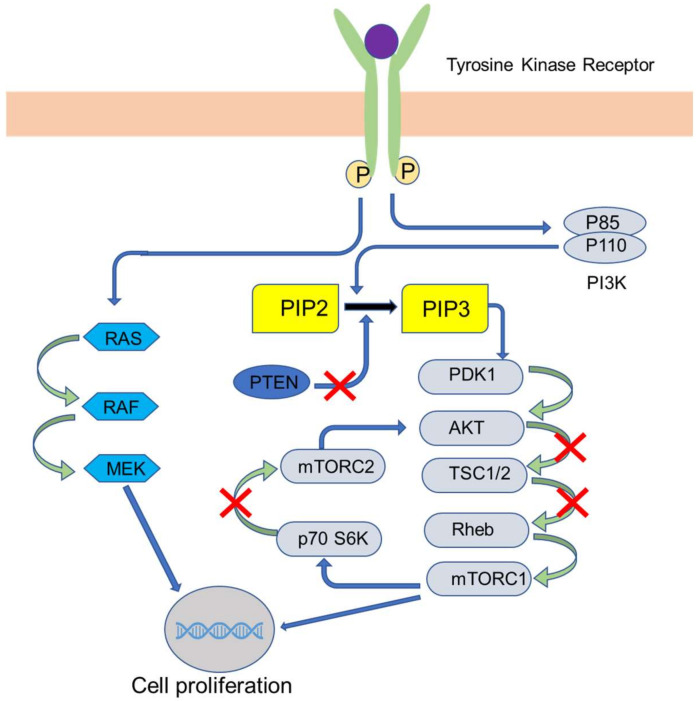
Schematic representation of the mechanism of the phosphatidylinositol 3-kinase (PI3K)/protein kinase B (AKT)/mammalian target of rapamycin (mTOR) pathway. PI3K—phosphatidylinositol 3-kinase, PIP—phosphatidylinositol–3,4,5-trisphosphate, PTEN—phosphatase and tensin homolog, PDK—phosphoinositide-dependent protein kinase, AKT—a serine/threonine-specific protein kinase, TSC—tuberous sclerosis protein, Rheb—Ras homolog enriched in brain, MTORC1—mammalian target of rapamycin complex-1, RAF—an acronym for rapidly accelerated fibrosarcoma, MEK—mitogen-activated protein kinase.

Cell growth, survival, motility angiogenesis, and metabolism are regulated by the phosphatidylinositol 3-kinase (PI3K)/protein kinase B (AKT)/mammalian target of rapamycin (mTOR) pathway [20]. Activation of this pathway leads to tumor development and drug resistance [21]. Over 70% of BC cases have shown activation of this pathway. Binding of growth factor to the G-protein-coupled receptor or the tyrosine-kinase receptor activates the PI3K–AKT–mTOR pathway [20]. The PI3K complex has p110α, which is a catalytic subunit, and p85, which is a regulatory subunit. The subclasses PI3K α, β, and δ are associated strongly in carcinogenesis promotion [22,23]. The mechanism of the PI3K–AKT–mTOR pathway is shown in Figure 3. The activation of this pathway leads to the phosphorylation of intracellular phosphatidylinositol-4,5-bisphosphate (PI-4,5-P2) to phosphatidylinositol–3,4,5-trisphosphate (PIP3), which is brought about by PI3K and later binds to pleckstrin-homology domains of AKT (a serine-threonine protein kinase) and other proteins [24]. Following this, AKT is activated on threonine residues (308 and 473 positions) induced by PI3. This AKT, in turn, activates mTORC1. mTORC1 is a complex molecule comprised of mTOR and protein, namely, Rheb, mLST8/GβL, PRAS40, and DEPTOR. The activated mTORC1 interacts with ribosomal protein S6 kinase (rpS6K) and eukaryotic translation initiation factor 4E1 binding protein (4E-BP1). These nuclear genes undertake the translation of genes responsible for cell proliferation and survival. Another complex, mTORC2, also contributes to AKT pathway activation via the phosphorylation of its serine residue at 473 position [20,22,25,26,27].

PI3K–AKT–mTOR pathway inhibitors can be classified as single inhibitors that block either AKT, mTOR, or PI3K and dual inhibitors that block two receptors, namely, mTORC1/mTORC2 and PI3K/mTOR [28,29,30]. The drugs, along with its mechanism of action on the receptors, are presented in Table 3.

The combination therapy constituting PI3K/AKT/mTOR inhibitors with other chemotherapeutic agents like paclitaxel and monoclonal antibodies like trastuzumab have shown promising results in HER-positive/HR-positive BC [31,32,33]. The drugs explored for the combination therapy, and newer drugs that are under clinical development, are summarized in Table 4.

##### Steroid Sulfatase Inhibitors

Steroid sulfatase enzyme is the chief cause of the conversion of inactive sulfate conjugated steroid into the active and free form [34]. It is found that estrogenic steroids may initiate the growth of cancerous cells in the breast. So, the best approach to arrest this is by inhibiting steroid sulfatase. The combination of irosustat with aromatase inhibitor therapy has shown promising clinical advantage with a satisfactory safety profile. However, the results suggested that a more detailed clinical study is essential to ascertain the success and to enable its effective clinical translation [34,35].

#### 2.2.2. Therapy for HER2 Positive Breast Cancers

##### PI3K/AKT/mTOR Inhibitors

PI3K inhibitors showed the best results in combination with lapatinib in treating patients suffering from HER2-positive advanced BC [36]. AKT inhibitors, along with trastuzumab, demonstrated significant antitumor activity [37]. The mTOR inhibitors, sirolimus [38] and ridaforolimus [39], along with trastuzumab, showed significant efficacy in HER2-positive BC therapy. However, activation of signaling pathways, including the PI3K/AKT/mTOR-pathway, has resulted in the development of resistance. The drug everolimus, an inhibitor of this pathway in combination with trastuzumab, has demonstrated a promising outcome in the advanced disease stage [40].

##### Monoclonal Antibodies (MAbs)

MAbs exert their action by targeting receptors and disrupting their signaling pathways. They target the cells via the immune system. Pertuzumab, trastuzumab, and margetuximab are the three MAbs currently available to treat BC, HER2-expressing tumors in particular. Trastuzumab acts by binding to the ectodomain of HER2 and blocks HER2 homodimerization, whereas pertuzumab acts by binding to subdomain-II of HER2 and prevents heterodimerization with HER3 [41]. Trastuzumab remains the mainstay of therapy for HER2-positive patients. As per the current treatment guidelines, trastuzumab, in combination with other chemotherapeutic agents, is recommended for a year. Pertuzumab, along with trastuzumab, in combination with additional chemotherapy, is recommended for patients with a high risk of cancer recurrence [42]. Margetuximab is a novel Fc-engineered HER2-targeted antibody that acts by the same mechanism as that of trastuzumab. It has a substitution of five amino acids at the IgG1-Fc (immunoglobulin class G1-crystallizable fragment) domain. Because of this modification, margetuximab has a higher affinity for Fc-receptors, which leads to increased antibody-dependent cell-mediated cytotoxicity [43]. As a single agent, margetuximab showed promising activity in phase I trial patients with HER2-positive MBC [44]. It has revealed a better overall response rate and longer median progression-free survival in the SOPHIA trial-phase III, which was conducted to compare margetuximab with trastuzumab, in combination with chemotherapy, in patients who have been previously treated with anti-HER2 targeted therapy (Clinical trial ID-NCT02492711). MAbs, along with other anticancer agents, has shown promising activity with reduced side effects, as compared to a single treatment. As cancer cells are continually undergoing mutation, there are higher chances that these may develop resistance to targeted therapy, such as MAbs. Cancer cells develop new checkpoint targets and antigens as they undergo mutation. Modern techniques like cancer genome sequencing can be utilized to study new targets for MAbs therapy, which may lead to the development of more effective MAbs [45].

##### Tyrosine Kinase (TK) Inhibitors

Neratinib, an irreversible pan-HER TK inhibitor, is used to reduce the chances of recurrence when given as adjuvant therapy in the early stage of HER2-positive BC. Neratinib is more efficient in blocking HER2 compared to trastuzumab, which may be the reason for its improved clinical outcome [46,47]. Neratinib alone (Clinical trial ID-NCT02673398) and in combination with T-DM1, capecitabine, and fulvestrant (NCT03377387, NCT02236000, NCT03289039) are currently under clinical development to evaluate the efficacy of these drugs. Neratinib has shown positive results in the NALA phase III trial (NCT01808573), which was conducted to assess the combined effect of capecitabine with neratinib versus capecitabine with lapatinib.

US FDA approved neratinib for the treatment of HER2 positive BC in Feb 2020 [48]. New TK inhibitors, namely, poziotinib, tucatinib, and pyrotinib, are presently under clinical development. Of these, pyrotinib and poziotinib, which belong to the irreversible pan-HER kinase inhibitor category, have shown significant toxicity in phase I and II trials [49]. Poziotinib, in the phase II trial, was administered as monotherapy after two lines of anti-HER2 therapy (namely, T-DM1, trastuzumab, pertuzumab, lapatinib), which showed activity by providing four months of median progression-free survival (PFS) [50]. Pyrotinib monotherapy under the same conditions has shown an overall response rate (ORR) and clinical benefit rate (CBR) of 50% and 61%, respectively [49]. Pyrotinib with capecitabine showed significant efficacy with ORR and median PFS of 78.5% and 18 months, respectively, compared to the combination treatment of capecitabine with lapatinib in patients with HER2-positive metastatic-BC [51]. Other clinical trials (NCT02659514, NCT02544997) are ongoing to investigate the efficacy of poziotinib in HER2-positive MBC. Tucatinib inhibits HER2 selectively, with least/no inhibition of EGFR. Tucatinib is currently in a phase Ib clinical trial in combination with trastuzumab or capecitabine or both. It has shown significant benefits of ORR and median PFS of 61% and 10 months, respectively, in pretreated HER2 BC patients [52]. Studies have revealed that tucatinib has the ability to cross the blood-brain barrier. Currently, it is under clinical trial (NCT02614794) in combination with capecitabine, along with trastuzumab with or without tucatinib, in patients with HER2-positive metastatic-BC, with or without metastasis of brain, after prior treatment with T-DM1, pertuzumab, and trastuzumab [53].

##### Antibody-Drug Conjugates (ADCs)

ADCs are the new class of prodrugs in which a cytotoxic drug is coupled with MAbs. ADCs are highly selective to tumor cells and can hence reduce systemic toxicity [54,55]. T-DM1, emtansine combined with trastuzumab, is the first ADC approved by the US FDA to treat HER2-positive BC. DS-8201a, an ADC comprising of deruxtecan, a topoisomerase-1 inhibitor coupled with HER2-targeting antibodies, showed an ORR and disease control rate of 64.2% and 94%, respectively, in a phase I clinical trial with HER2-positive and HER2-negative low metastatic-BC patients [56]. Two different phase III clinical trials to study the efficacy of DS-8201a are currently under investigation: NCT03523585, designed for the comparison of DS-8201a with capecitabine, trastuzumab, and lapatinib in HER2 BC patients treated with T-DM1 previously, and NCT03529110, which compares the efficacy and safety of DS-8201a and T-DM1 in the HER2 BC patients treated with taxane [57]. SYD985 is another novel ADC developed by conjugating duocarmazine with trastuzumab via a linker. SYD985, when compared with T-DM1, showed a greater antitumor effect in preclinical studies [58], with encouraging results in a phase I clinical study [59]. NCT03262935, a phase III study in HER2-positive BC patients, wherein SYD985 is being compared with the physician’s current treatment choice, is presently being investigated. On similar lines, two other ADCs, namely, XMT-1522 (auristatin conjugated with anti-HER2 antibody) and RC48 (E-derivative of auristatin conjugated with anti-HER2 MAb hertuzumab) are currently under phase I clinical trials, which have shown a good CBR [60,61]. Efforts were made to target two different epitopes of HER2 (bi-specific ADC MEDI4276), which failed to show a positive outcome in phase I and, hence, was out of the race [62].

##### Immunotherapy

Immunotherapy has recently emerged as one of the promising therapeutic approaches for BC. Many studies have been undertaken in this regard; for example, a recombinant molecule (HER2-Fc) was developed for targeting human dendritic cells. This recombinant protein has shown to provoke the immune response of T-cells against HER2 positive BC [63]. CD8 positive T-cell vaccines are shown to have significant anti-breast cancer activity. Preclinical data revealed that trastuzumab amplified the chances of tumor cell death by the vaccine CD8 positive T-cells, giving a hint to go ahead with the study. The study was carried out on healthy subjects, human leukocyte antigen A2-positive and A3-positive, and HER2-positive BC patients. Healthy subjects were vaccinated with trastuzumab, and patients were administered with a monthly dose of GP2-positive granulocyte-macrophage colony-stimulating factor (GM-CSF), followed by trastuzumab, for the next six months. In addition to the left ventricular ejection fraction (LVEF), local and systemic toxicity was observed. The outcome of the study revealed that the GP2-positive GM-CSF vaccine was found to be safe to be administered along with trastuzumab, and they were found to stimulate an immunologic response [64].

#### 2.2.3. Therapy of TNBC

TNBC is the most aggressive form of breast cancer with higher rates of recurrence. Two genes, namely, BRCA1 and BRCA2, are said to be associated with TNBC. These genes are involved in the repair of DNA abnormalities, which lead to cancer development and the uncontrolled growth of the tumor. Hence, they are also called tumor suppressor genes [65,66]. A mutation or defect in the BRCA gene leads to the stoppage of its activity of repairing DNA sequence and preventing cancer, which leads to the development of TNBC [67]. Around 55–65% and 45% of women with BRCA1 and BRCA2 mutations, respectively, will develop breast cancer. The recurrence of breast cancer in BRCA-mutated patients after first-time treatment is higher than average [66]. The patients with a BRCA1 mutation are more likely to develop TNBC and high tumor burden when compared to patients with BRCA2-mutated genes [66,68]. Approximately 5% of TNBC patients have a BRCA mutation, out of which 40–50% of the patients are BRCA1 mutated [69,70]. The patients with BRCA1-mutated TNBC show a higher number of tumor-infiltrating lymphocytes, high programmed cell death protein-1 (PD-1) and cytotoxic T-lymphocyte-associated protein 4 (CTLA-4) expression when compared with BRCA-1 nonmutated TNBC [71]. As cancer cell growth does not depend on hormones in TNBC, patients are unlikely to respond to hormone therapy, and hence, alternate options such as surgery and radiation along with chemotherapy are being adopted [72,73].

Immune checkpoint inhibitors (antibodies against PD-1 and CTLA-4) with cisplatin have been explored for the treatment of patients suffering from TNBC with a BRCA1 mutation. This has shown significant shrinkage of tumors and a high survival rate. Novel targeted therapies using poly (ADP-ribose) polymerase (PARP) inhibitors such as talazoparib, olaparib, and niraparib, in combination with immune checkpoint inhibitors, have shown positive clinical outcomes in patients [74,75]. Many other drugs that have additional immunotherapy benefits, along with their cytotoxic activity, are widely used for the treatment of TNBC, namely, anthracyclines, taxanes, cyclophosphamide, gemcitabine, and platinum salts. Anthracyclines activate dendritic cells and specific T-cells and increase CD8 T-cells, which are instigated by the apoptosis of cancer cells caused by anthracyclines [76]. The patients treated with taxanes have shown an increase in the number of tumor-infiltrating lymphocytes (TIL) [77]. They partially reduce the immunosuppression in the microenvironment of the tumor by selectively decreasing myeloid-derived suppressor cells (MDSCs) and regulatory T-cells [78,79,80]. Cyclophosphamide exerts its action by suppressing regulatory T-cells, inducing immunological cell death, and increasing the proliferation of natural killer and CD8 T-cells [81,82]. Gemcitabine helps in increasing the CD8 T-cells’ anti-tumor activity and can also reduce MDSC counts [83,84], while platinum salts promote T-cell activation and induce immunologic cell death [85,86,87].

##### Poly (ADP-ribose) Polymerase (PARP) Inhibitors

Owing to its heterogeneity, the diagnosis and treatment of TNBC is challenging. Standard chemotherapy, comprising of a combination of a drug containing platinum, is recommended by the physicians. PARP inhibitors are the most novel, promising agents that have shown positive outcomes in both BRCA-associated and sporadic TNBC. Carboplatin and PARP inhibitors, when used in combination, can target the impaired DNA repair pathway in patients with BRCA1/2 mutations. PARP and BRCA1/2 have the basic difference of repairing ssDNA (single-stranded deoxyribonucleic acid) breaks and dsDNA (double-stranded deoxyribonucleic acid) breaks, respectively, through homologous recombination [88]. Olaparib, a PARP inhibitor, in phase III clinical trial, has shown the lowered risk of death and an improved median of 42% and 2.8 months when compared with standard chemotherapy [69]. Talazoparib (BMN 673), which is currently in phase II clinical trials (NCT01945775), has shown significant benefit as a single agent when compared with standard chemotherapy in germline BRCA1/2-mutated advanced-BC patients. The ability of it to trap the PARP–DNA complex by actively binding to the DNA makes it clinically significant [89]. Other PARP inhibitors such as veliparib (in phase III clinical trial, NCT02163694), niraparib (in phase III clinical trial, NCT01905592), and rucaparib (in phase II clinical trial, NCT02505048) are under investigation in germline BRCA1/2-mutated and advanced-BC patients. The responsiveness of the TNBC to PARP inhibitors or platinum therapy is based on three DNA-based homologous recombination deficiency scores, which reflect BRCA1/2 genetic defects [90].

##### Anti-Angiogenic Agents

Vascular endothelial growth factor (VEGF), an angiogenic factor, is one of the significant markers in TNBC. VEGF is expressed in the intratumoral region, and to treat this subtype, bevacizumab, a MAb, is used. These antibodies act by inhibiting metastasis and suppressing tumor neovasculature. According to the results of preclinical studies, bevacizumab, when added with docetaxel (a first-line chemotherapy drug), showed a significant response rate in the patient [91,92]. Bromodomain extraterminal (BET) proteins coordinate transcription by identifying specifically acetylated lysine on chromatin. BETi (BET inhibitor) competes with the procedure of suppressing the gene expression. Hypoxia allows the progression of TNBC. Thus, BETi, along with JQ1 (a thienotriazolo diazepine and potent inhibitor of BET family, named after its developer Jun Qi), were studied to express anticancer activity. JQ1 was successful in modulating cancer activity by downregulation, together with CA9 and VEGF-A. In addition, JQ1 prevented hypoxia-inducible factor (HIF) binding to the hypoxia response element in CA8. JQ1 has also been reported to reduce TNBC growth both in vitro and in vivo, and, therefore, JQ1, along with BETi, have demonstrated good antitumor activity [93].

##### Immunotherapy

PD-1 and PD-L1 (PD-ligand1) are the proteins present on T-cells and healthy cells (and more in number on cancer cells), respectively. PD-1, a programmed cell death receptor, is involved in the suppression of T-cells. They accomplish this by inhibiting the auto-immune response of T-cells and prevent them from acting against killer cancer cells [94]. Atezolizumab, an anti-PD-L1 antibody along with Nab-paclitaxel (Abraxane^®^), was approved by the US FDA in 2019 for unresectable locally advanced or metastatic TNBC [95]. Trials are currently underway in patients with a positive PD-L1 response, for example, pembrolizumab, a human MAb that has demonstrated durable anticancer activity [92,96,97]. In another study, an mRNA vaccine incorporated into nanoparticles (NPs) to act against tumor antigen MUC1 on dendritic cells (DCs) in lymph nodes was developed. The anti-CTLA-4 (cytotoxic T-lymphocyte associated protein-4) monoclonal antibody was added to the mRNA vaccine to increase its activity against cancer. In vivo studies were carried out, which showed that the vaccine targeted mannose receptors on DCs and exhibited anticancer activity against TNBC 4T1 cells. It also showed that the combination therapy had an additional antitumor activity, rather than the vaccine alone [64].

##### Monoclonal Antibody

MAbs are emerging as an exciting alternative for the treatment of TNBC. A protein, glycoprotein NMB (transmembrane glycoprotein), is overexpressed in TNBC. This protein is related to the invasion of the tumor and cell metastasis, as it is a transmembrane protein. A monoclonal antibody-cytotoxic drug (glembatumumab vedotin) was formulated to target this protein directly, and this trial has shown positive results in cancer treatment [98]. Inhibition of PD-1 or PD-L1 has demonstrated anticancer activity in treating metastatic breast cancer (MBC). Researchers measured the response of the drug avelumab, a PD-L1 inhibitor, in patients affected with MBC. In phase I studies, patients received standard intravenous avelumab therapy every two weeks. Tumors were observed for every six weeks, and immunohistochemistry was performed to examine the expression of the membrane PD-L1. In a subset of MBC patients, avelumab showed a tolerable safety profile and clinical activity [99]. Table 5 presents a list of drugs for TNBC therapy that are currently under clinical investigation.

##### RANKL and RANK System

**Figure 4:**
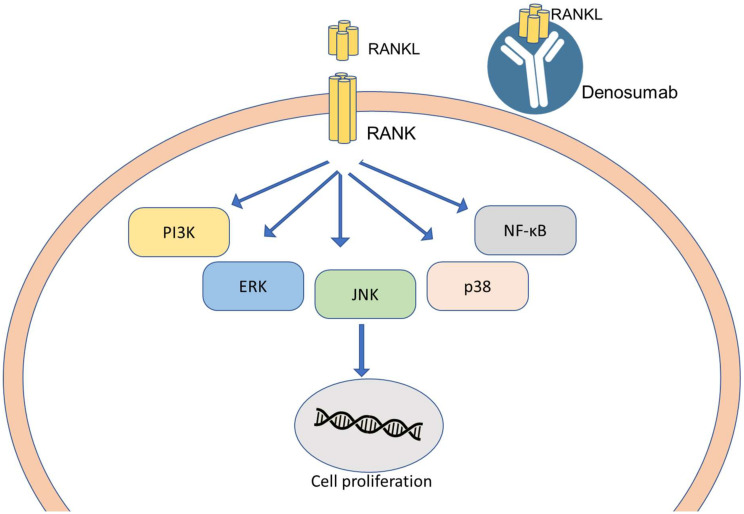
Schematic representation of the RANK/RANKL pathway. Pathways activated by RANK-RANKL signaling and downstream targets. ERK = extracellular signal-related kinases; JNK = cJun amino-terminal kinases; NF-κB = nuclear factor κB; PI3K = phosphoinositide 3-kinase; RANK = receptor activator of nuclear factor κB; RANKL = receptor activator of nuclear factor κB ligand; TRAF = TNF receptor-associated factor.

The tumor necrosis factor (TNF) is involved in various cellular events such as cell differentiation, proliferation, survival, and death. Inflammatory cells secrete the TNF is a type of inflammation-associated carcinogenesis [100]. RANKL (receptor activator nuclear factor-κB ligand) is a member of TNFα that binds to RANK (receptor activator of nuclear factor-κB), a membrane receptor. The binding of RANKL to RANK receptor promotes the proliferation of mammary epithelial cells and development of tubuloalveolar mammary tissue [101]. RANK promotes tumorigenesis in epithelial cells of breast tissue by causing a transition in epithelial-mesenchymal cells. Along with the hormone-dependent RANK/RANKL effect, RANKL is also capable of forming breast tumors, followed by metastasis [102]. Identification of this pathway and its role in breast cancer development has paved the way for its possible utilization as a potential target for breast cancer therapy. The mechanism is presented in Figure 4. The RANK/RANKL pathway can be blocked by using an antibody that can selectively block RANK and prevent the attachment of RANKL to RANK receptors, thereby preventing the activation of RANK. Denosumab, a monoclonal antibody for RANK at present, is used for treating osteoporosis [103]. Blocking the RANK/RANKL pathway also reduces the risk of BC development in postmenopausal women [102,104]. Studies are still underway to define the mechanism of this pathway in BC development, which, if proved, could be a novel target.

## 3. Multidrug-Resistant (MDR) BC

Resistance to BC therapy is one of the major obstacles that limit the effectiveness of the treatment in BC patients, leading to recurrence. Multidrug resistance refers to a condition where cancer cells develop cross-resistance to chemotherapeutic drugs due to a multitude of factors. Some of the patients develop resistance to certain medications before treatment, which may be attributed to their genetic makeup and is referred to as intrinsic drug resistance. Acquired resistance is a kind of resistance that a patient develops over a period of time following exposure to the treatment. This resistance is the main reason for the relapse of cancer even after the treatment or during the treatment. The main mechanisms of drug resistance are increased efflux of drugs, alteration of drug target, enhanced DNA damage repair, senescence escape, epigenetic modifications, tumor heterogeneity, changes in the tumor microenvironment, and epithelial-mesenchymal transition [105,106].

Valspodar (PSC833) is a P-glycoprotein inhibitor that plays a significant role in efflux-mediated MDR that has shown promising results by increasing mitoxantrone accumulation by 94% when administered before treatment with mitoxantrone [107]. Paclitaxel has shown improved effectiveness against MDR-BC in patients who were administered valspodar before treatment than those without valspodar administration (ClinicalTrials.gov Identifier: NCT00002826). Drugs such as docetaxel, laniquidar, and paclitaxel, when given in combination, have shown to combat MDR-BC in patients with metastatic-BC (ClinicalTrials.gov Identifier: NCT00028873). Photodynamic therapy is another novel strategy that has been explored and has proved to reverse MDR, possibly by inhibiting the pathways that are involved in MDR [108]. Lu Yang and co-workers in their studies showed that PEPDG278D, a recombinant peptidase, which has a strong affinity for the HER2-receptor, inhibits the same receptor. Preclinical studies conducted revealed high activity in trastuzumab and other anti-HER2-resistant BC [109]. Ritonavir, an antiretroviral drug, was shown to overcome drug resistance when administered along with other anticancer agents due to its ability to deactivate metabolizing enzymes and efflux transporters, which are overexpressed in MDR-BC [110].

## 4. Nanomedicine Used in the Management of Breast Cancer

Nanomedicine is a rapidly emerging field in the treatment of various diseases. It has shown promising results in cancer therapy by offering new alternatives to the currently existing diagnosis and therapies. Numerous nanoparticles have been developed with targeting properties towards cancer cells, along with metastatized breast cancer [111]. The nanoparticle formulations that have been approved are listed in Table 6.

Nanoparticles (NPs) offer many promising properties to active pharmaceutical ingredients, together with longer elimination time, increased drug–target interaction time, and reduced drug resistance. The advantage of NPs is increased drug interaction with the cancer-affected area and minimized drug resistance. The nanoparticle drug transporters encompass a minimum of two ingredients, one being the active pharmaceutical ingredient and the other being the carrier. NPs signify multipurpose tools to encapsulate a variety of medicine and can be custom-tailored for patients by simple conjugation methods [111,112,113]. Further subclassification is discussed in the following sections. The nanocarriers that have been explored for the treatment of BC are presented in Table 7.

### 4.1. Polymer-Based Nanoparticles (PBNP)

NPs containing polymer as an integral ingredient are known as polymeric NPs, which can be of either natural or artificial origin. Polymer-based nanoparticles (PBNPs) have the advantage of sustained drug delivery, long blood-circulation time, and can also be tuned for pH-, radiation-, and temperature-responsiveness. When it comes to drug loading into the NPs, it can be done by two processes: the first being physical entrapment, and the second is covalent linking to the compound matrix [111]. Researchers have targeted essential genes responsible for the multiplication of cancerous cells. To achieve this goal, siRNA (small interfering RNA) was designed, which were delivered in vivo to inhibit the growth of cancerous cells. Here, a mesoporous silica NP core was loaded with crosslinking polyethyleneimine-polyethylene glycol copolymer loaded with siRNA to target HER2 oncogene. It was coupled with trastuzumab, a monoclonal antibody that increases the blood half-life of siRNA. Along with this property, specific uptake of nanoparticles by cancerous cells was also increased. It was observed that a single dose of siRNA nanoparticles showed a 60% decrease in HER2 protein levels in a trastuzumab-resistant xenograft. The study was continued for three weeks and showed significant inhibition in the growth of tumor cells. The prepared siHER2 NPs (siRNA NPs against HER2 designated siHER2 NP) showed a better safety profile and negligible cytokine induction in comparison to peripheral mononuclear cells [140]. In another study, poly(lactide-co-glycolide), poly(lactic acid), and poly(ε-caprolactone) nanoparticles loaded with anastrozole, an anti-cancer drug, were developed. The prepared formulations were tested for its in vitro cytotoxicity and in vivo pharmacokinetics in rats. The entrapment efficiency of the formulations ranged between 35–85%, which depended on the drug-polymer ratio. The area under the curve for nanoparticles prepared using three different carrier systems was higher when compared with anastrozole in solution. The pharmacokinetics of these polymeric nanoparticles have revealed the extended circulation of drugs and have proved effective in breast cancer chemotherapy [113,141]. In another study, the use of polymeric nanoparticles for TNBC therapy was explored using chitosan/polylactide nanoparticles loaded with tamoxifen, which showed significant activity against TNBC cells, causing cell death and cell arrest [142]. Researchers have explored PBNP not only for drug loading but also for theranostic purposes. Jin and co-workers conjugated luminescent poly[2-methoxy-5-(2-ethylhexyloxy)-1,4-phenylenevinylene] with the cyclic arginine-glycine-aspartic acid conjugated polymer nanoparticles, which generates reactive oxygen species (ROS) when irradiated with light and has shown promising results on MDA-MB-231 cells [143]. Nuannuan Li and co-workers synthesized pH-sensitive self-assembling amphiphilic copolymers formulated into polymersomes by incorporating verapamil hydrochloride and doxorubicin hydrochloride, to overcome doxorubicin resistance in the MCF-7/ARD cell line [144]. Yao and co-workers fabricated multifunctional pH-responsive nanocarriers for the delivery of anticancer drugs that constituted poly(2-(diisopropylamino)ethyl methacrylate) (PDPA) polymer as the inner core and amphiphilic lipid-poly(ethylene glycol) (lipid-PEG) as the outer shell, conjugated with iRGD peptide on the surface. The studies have suggested the plausible use of this novel platform for the effective treatment of cancer [145].

Micellar nanocarriers, another set of self-assembling colloidal systems comprising of a hydrophobic core with a hydrophilic shell made up of surfactant and hydrophilic polymers, have also been explored for breast cancer therapy. A new redox-sensitive micellar system consisting of hyaluronic acid (HA)-based amphiphilic conjugate (HA-ss-(OA-g-bPEI), HSOP) was formulated for tumor-based codelivery of paclitaxel (PTX) and AURKA-explicit siRNA (si-AURKA) for the treatment of targeted cancer therapy. HSOP showed outstanding loading for both PTX and siRNA, with regulating dosage ratios and necessary redox sensitivity individually. This was confirmed by morphological fluctuations of micelles with the in vitro release of both drugs in different reducing environments. The HSOP micelles were able to deliver both PTX and siRNA simultaneously into MDA-MB-231 BC cells via HA-receptor mediated endocytosis, followed by quick transport into the cytosol, which was confirmed by flow cytometry and confocal microscopic investigation. The synergistic effects between the drugs were enhanced by the specific delivery and transport when compared to single drug-loaded micelles and nonsensitive coloaded micelles, leading to significantly higher antitumor efficacy. The in vitro examination found that HSOP micelles could accumulate effectively in cancerous sites and had the maximum antitumor effectiveness over nonsensitive control of codelivery and redox-sensitive single-drug controls. The redox-sensitive HSOP codelivery system is promising in combining drug/gene therapy for targeting tumor therapy [146]. Studies have shown that the TNBC cells that are resistant to conventional chemotherapy can be photomechanically killed, with few laser beams using bioinspired-NPs [147]. Xie and co-workers formulated polymeric hybrid-nanomicelles for the codelivery of DOX/MiR-34a, with high tumor suppression and enhanced MRI in a BC mice model [148]

### 4.2. Lipid-Based Drug Carriers

Lipid-based NPs are the colloidal systems comprising of biocompatible lipid cores. These systems have the advantages of high biocompatibility, thermal stability, drug-loading efficiency, and sustained drug release. The lipid-based carriers include liposomes, solid lipid nanoparticles, and nanostructured lipid carriers. These carriers have the ability to transport hydrophobic as well as hydrophilic drugs with low toxicity.

Liposomes are the self-assembling lipidic nanoparticles consisting of a lipid-bilayer. These are novel carrier systems available in the marketplace for the treatment of cancer. A major obstacle in the successful treatment of cancer is the multidrug resistance (MDR) offered by cancerous cells. In this regard, two drugs, namely, resveratrol, and paclitaxel, were coencapsulated with a PEGylated liposome as a combinational therapy. The encapsulated liposome showed 50% encapsulation efficiency and an improved in vitro cytotoxic activity against a drug-resistant MCF-7 cell line. Along with this property, it also demonstrated improved bioavailability and drug retention in tumor cells. When administered to mice, it effectively worked against drug-resistant tumors, with no prominent systemic toxicity. The study thus proposed that codelivery of resveratrol and paclitaxel in a nanocarrier system may present an enhanced therapeutic efficiency against MDR cancerous cells [149]. Another study was performed wherein PEGylated liposomes loaded with anastrozole were prepared by the film hydration method for the effective treatment of breast cancer. The liposomes were prepared using soyaphosphatidylcholine, cholesterol, and methoxy polyethylene glycol distearoyl ethanolamine. The optimized formulations were assessed for their activity by in vitro cell line studies on BT-549 and MCF-7 cell lines and for their in vivo behavior in Wistar rats. The area under the curve (AUC) showed a many-fold increase in the case of PEGylated liposomes, which substantiated that PEGylated liposomes could improve systemic circulation and deliver a sustained release of drugs for breast cancer therapy [150]. Recently, ruthenium(III)-complexes containing liposomes demonstrated promising results in treating different types of BC, including TNBC, by causing sustained cell death [151]. In another study, antibody-tethered liposomes loaded with docetaxel, a prodrug targeting Ephrin receptor A2, were developed, which showed reduced drug concentration in systemic circulation by achieving the required drug concentration at the tumor microenvironment in a sustained manner, thereby reducing systemic side effects [152]. Liposomes formulated by loading imiquimod R837, an immune adjuvant TLR7 agonist, and hematoporphyrin monomethyl ether, a sonosensitizer, showed promising tumor growth arrest and was also helpful in preventing metastasis to the lung [153]. The US FDA has approved formulations containing liposomes of which Doxil^®^ (pegylated liposomal doxorubicin) is the first FDA-approved (1995) ‘*nanodrug’* for the therapy of metastatic ovarian cancer and AIDS-related Kaposi’s sarcoma. Table 8 lists the liposomal-based formulations that have been approved by the US FDA and are under clinical development.

Solid lipid nanoparticles (SLNs) are the colloidal systems consisting of a solid lipid matrix, which were developed as an alternative to traditional emulsions and liposomes. These carriers have also been exploited for plausible use in breast cancer therapy. Researchers have developed letrozole-loaded SLNs with folic acid surface modification, which presented significantly higher cytotoxicity when compared with the free drug in the MCF-7 cell line by caspase-3 dependent apoptosis [154]. Abd-Ellatef and coworkers formulated curcumin-loaded SLNs coated with chitosan and tested them on doxorubicin-resistant breast cancer cells. There was a 5 to 10-fold increase in the toxicity and intracellular retention of doxorubicin in TNBC cells resistant to doxorubicin [155]. These SLN carriers were further improvised by mixing liquid lipid and solid lipid in a particular ratio to form a lipid core, which has helped in overcoming drug leaching during storage; these are named nanostructured lipid carriers (NLCs). These NLCs have several advantages over the conventional carriers in terms of prolonged half-life, increased permeability, biocompatibility, and improved storage stability. These NLCs have also been exploited for the loading of drugs for the treatment of breast cancer. In one such study, lapachone and doxorubicin were coloaded in NLCs and assessed for the treatment of MDR BC. In vitro studies on MCF-7 ADR cells showed increased uptake of doxorubicin when given in combination. In in vivo studies on MCF-7 ADR, tumor-bearing mice revealed enhanced efficacy when administered in combination, as compared to doxorubicin monotherapy [156]. Phytochemical resveratrol also showed improved efficiency when loaded into NLCs tethered with folic acid [157]. In another study, efforts were made to improve the targeting efficiency of paclitaxel and photosensitizers to tumor cells. A novel carrier containing paclitaxel, chlorin e6 conjugated with folic acid, was formulated to target tumor tissues. In vitro cell line studies on MDA-MB-231 cells showed an enhanced uptake that was substantiated by the in vivo studies on nude mice, which revealed that the combination of anticancer drugs and photodynamic therapy had an improved outcome in the reduction of tumor size [158]. In yet another study, the efficacy of NLCs loaded with epigallocatechin gallate and protamine sulfate, functioning as a stimuli-responsive agent to the presence of ATP, was evaluated for BC therapy. The results of these studies revealed promising outcomes in the treatment of BC [159].

### 4.3. Dendrimers (DM)

Dendrimers are highly branched macromolecules with a polymeric core, interior branches, and a functionalized exterior surface. They are multipurpose, changeable structures with a monodisperse size and can be easily loaded with different therapeutic agents. Dendrimer-modified magnetic NPs have shown an enhanced therapeutic value of chemotherapy. They have proved to be a useful source of magnetic resonance (MR) image contrast agents [160]. Researchers have prepared curcumin-loaded dendrimer-based ferric oxide nanoparticles coated with citric acid. These NPs were developed using polyamidoamine (PAMAM), a generation 5.0 dendrimer. To assess the antitumor activity of the prepared formulation, curcumin-alone and curcumin-loaded nanocarriers were compared by using a 3-(4,5-dimethylthiazol-2-yl)-2,5-diphenyltetrazolium bromide assay on MCF-7 cell lines. The results revealed that the dendrimer-modified NPs proved more effective against cancerous cells as it showed a slow and controlled release of drugs in the treatment of BC [161]. In another study, docetaxel-loaded dendrimers that were grafted with trastuzumab on the surface were synthesized. Trastuzumab-grafted DM showed higher internalization and antiproliferative effects on MDA-MB-453 than that of DM without trastuzumab grafting [162]. The plausible role of epidermal growth factor receptor (EGFR)-binding peptide-1 (EP-1) as a targeting agent against TNBC was assessed using poly(amidoamine) DM coupled with EP-1 and transactivating transcriptional cell-penetrating peptide-loaded with doxorubicin. The studies revealed that this DM acted as a dual functional drug carrier and showed a high antiproliferative effect on a TNBC MDA-MB-231 cell line when compared with free doxorubicin and DM without grafting [163]. Scientists have conjugated methotrexate with poly(amidoamine) DM, which showed two-times higher efficacy than that of plain methotrexate [164]. A study was conducted to test and demonstrate that the nanoscale system based on the Janus (a peptide) dendron drug conjugate can effectively act as a vehicle for the delivery of chemotherapeutic drugs for BC. The Janus dendron was fruitfully reformed with mPEG and PVGLIG-doxorubicin (DOX), through 2 steps, very effectively by cycloaddition (CuAAC) copper-catalyzed alkyne-azide click. The developed nanosystem showed significantly fewer side effects and higher efficiency, proving it to be a potent chemotherapeutic delivery vehicle for BC when compared to free DOX [165].

### 4.4. Aptamer

Aptamers are peptides or oligonucleotides that have the ability to bind to specific targets [166]. They have a unique pharmacokinetic profile, which makes their formulation difficult when compared to other proteins. Researchers are working on it and have come up with a new methodology to formulate a transducer on a gold surface, which provides a larger surface area to restrain the high quantity of the aptamer. The apta-sensor was deployed to detect the platelet-derived growth factor (PDGF) using square wave voltammetry (SWV) and cyclic voltammetry (CV) techniques. The proposed GNPs-cubic-α-CD-Apt-Au electrode apta-sensor displayed excellent analytical performance for MCF-7 cell determination under optimized experimental conditions. The electrochemical apta-sensor could detect cancer-related targets with much higher efficiency in unprocessed human plasma samples [167]. Efforts were made to overcome drug resistance by developing liposomes loaded with doxorubicin-aptamer AS1411 (Ap-Dox). In vitro studies on MCF-7/ADR cells revealed that the liposome carrier tagged with aptamer was capable of getting bound to nucleolin and effectively caused cell death [168]. In another study, paclitaxel-loaded PLGA nanoparticles functionalized with PEG were developed, which were further conjugated with heparanase aptamer. In vitro studies on MDA-MB-231 cells revealed higher cell toxicity as compared to that of the nonfunctionalized carrier. Preclinical studies have shown a significant reduction in tumor volume compared to that of plain nanoparticles, suggesting that heparanase aptamer may be a promising target for TNBC drug delivery [169]. Wang and co-workers formulated an aptamer surface modified with the nanotags of surface-enhanced Raman scattering agents. These aptamers demonstrated precise imaging capability with high toxicity on irradiation with lasers, due to their photothermal property, in the MCF-7 cell-lines [170]. Mesoporous silica on top of iron nanoparticles loaded with doxorubicin (DOX) and tethered with mucin-aptamer on the surface demonstrated MRI ability and high cytotoxicity in mucin overexpressed BC cell lines [171].

### 4.5. Inorganic Nanoparticles

**Figure 5:**
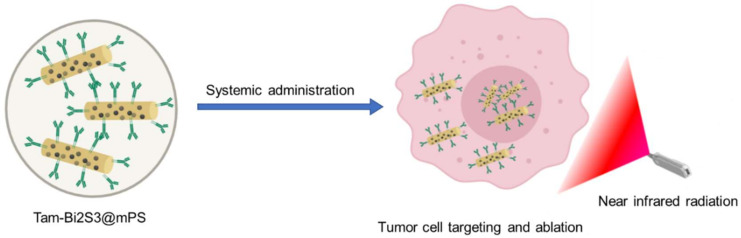
Diagrammatic presentation of the targeted Tam-Bi2S3@mPS theranostic nanoplatform.

Of late, inorganic particles have been gaining widespread interest and have been widely explored in the diagnosis and treatment of cancer. They have advantages over organic nanoparticles in terms of their specific targeting ability and unique characteristics, which can be easily manipulated to target tumors [172]. There has been a clinical limitation of methotrexate (MTX) standalone treatment because of an extremely short plasma half-life. To combat this, a new system of drug delivery was proposed wherein an MTX-LDH (methotrexate-lactate dehydrogenase) nano-hybrid system was developed, which induced enhanced apoptosis when compared with free MTX. The annexin-V and propidium iodide dual bonding and tunnel investigation exhibited superior results and better targeting when investigated in mice bearing orthotropic human breast tumors, resulting in a greater reduction in tumor volume compared to standalone methotrexate after treatment of 2 h [173]. Xu and team studied the efficacy of pH, glutathione, and hyaluronidase triple-responsive gold nanoparticles as a therapeutic approach in CD44 and HER2 overexpressed BC. In addition, the developed nanoparticles also had the properties of imaging-guided photodynamic therapy (PDT) and photothermal therapy. When conjugated with 5-aminolevulinic acid (ALA), Cy7.5, and anti-HER2 antibody onto hyaluronic acid (HA) moiety, the nanoparticles showed enhanced cellular uptake and efficient cell death when compared to individual therapy. The in vivo results showed the elimination of cancer cells entirely, with minimal side effects, which suggest that the developed carrier can be efficiently utilized for the treatment of BC [174]. In another study, redox-responsive nanogels, conjugated with hyaluronic acid loaded with doxorubicin, were fabricated for the treatment of cancer. Gold nanorods, conjugated with hyaluronic acid-cystamine as a crosslinker, were developed and found to release the drug in response to the presence of intracellular glutathione. Studies on MCF-7 cell lines revealed enhanced uptake, whereas MCF-7 ADR cells showed reduced uptake due to drug efflux. However, on near-infrared irradiation, the resistance of the MCF-7 ADR cells could be overcome [175]. The concept of siRNA, which has the ability to silence genes, has greater applicability in patients with BRCA1/BRCA2 mutations. siRNA alone may not help to treat BRCA1/BRCA2-positive BC. The study on BRCA1-expressed HeLa cells demonstrated increased sensitivity and apoptosis to proteasome inhibitors such as carfilzomib and bortezomib after treatment with siRNAs [176,177]. A study conducted by Shenda and co-workers showed promising results in the reversing/overcoming of trastuzumab and lapatinib resistance in 18HER2-positive cell lines, wherein they formulated mesoporous silica loaded with siRNA and tagged with trastuzumab. The study also showed that cell lines did not develop resistance to long-time siRNA treatment [178]. siRNA (of survivin and CDC20)-loaded polyplexes, formulated using methylcellulose, hyaluronic acid, dextran sulfate, and polyacrylic acid, showed increased antiproliferative and cell death in different cell lines such as MDA-MB-436, SUM149PT, MDA-MB-231, and MCF-7 [179]. Gao and co-workers synthesized polydopamine coated titanium nanoprobes containing chlorine e6 chelated with manganese (II) ion. These multifunctional NPs have shown a synergistic effect of photothermal/photodynamic properties and magnetic resonance imaging (MRI) in the BC mouse model [180]. Targeted image-guided therapy of HER2-positive BC was developed using a theranostic agent that consisted of bismuth sulfide mesoporous silica NP core shells. The formulating procedure included chemical encapsulation of mesoporous silica with polyvinylpyrrolidone decorated rod-like bismuth sulfide mesoporous silica. The mesoporous layer consisted of doxorubicin. The NPs formed were chemically conjugated with a monoclonal antibody (trastuzumab), giving rise to trastuzumab bismuth sulfide mesoporous silica nanoparticles (Tam-Bi2S3@mPS). The formulation demonstrated improved drug loading capacity, biocompatibility, and specific and active cancer cell targeting, with enhanced accumulation inside the cells. They served two purposes: First, as an exceptional contrast development probe owing to the presence of bismuth for computer tomography for deep inside tumor tissue imaging. Secondly, it was observed that these NPs effectively destroyed tumors and inhibited metastasis by synergistic chemotherapy (Figure 5) [181].

### 4.6. Carbon Nanotubes (CNTs)

Aromatic hydrocarbon rings involved in the formation of carbon cylinders are known as carbon nanotubes. The solubility issues associated with its formulation can be resolved by linking it to proteins and peptides, which are water-soluble ligands. Therapeutic agents are linked to these water-soluble ligands to improve their targeted action when compared to the pure drug alone. In a study, the restraint of currently available treatments for BC was explained by cancer stem cells (CSCs), which play a crucial role in carcinoma instigation, advancement, reappearance, and metastasis. Thus, carbon nanotubes have emerged as a potential field for treating BC. Single-walled carbon nanotube (SWCNT) biocompatible multimodality nano samples were prepared, and the therapeutic efficacy and biodistribution of the CD44 antibody combined with SWCNTs were examined using different methods such as magnetic resonance imaging (MRI) and computed tomography for single-photon emissions. SWCNT quantification was performed by sensitively calculating iron content in sorted CSC populations, adopting inductively combined plasma-mass spectrometry, and the study established the improved targeting of formulated nanotubes. Immunohistochemistry analysis was performed, which showed enhanced localization of nanotubes in areas that had abundant CD44 receptors. These data provided inspiring outcomes effectively aimed at breast CSCs [182]. Researchers have conjugated CNTs with PEG and β-estradiol and loaded with lobaplastin. β-estradiol, being the estrogen receptor agonist, helps in targeting the estrogen receptor, and PEG helps in enhancing systemic circulation. The results revealed that the adverse effect on vital organs like the liver, heart, and kidneys was negligible, and the formulation exhibited sustained-release on the mice-BC model [183]. Singh and co-workers formulated multiwalled CNTs conjugated with thiamine and riboflavin loaded with paclitaxel, which showed promising results on MCF-7 BC cell lines [184].

### 4.7. Nanoshells (NSs)

Nanoshells (NSs) are optically active NPs, which, when injected systemically, mount up within the cancerous cells due to the stimuli-responsive enhanced permeability and retention (EPR) effect. They induce photothermal death of the tumor cells once irradiated with the near-infrared optical device [185]. Programmed mesoporous nanoshells capped with silica gold nanorods and coated with nano-selenium layer nanoparticles were formulated. These NPs behaved as multifunctional nanoplatforms to include materials with explicit chemotherapeutic activity, which contribute to enhancing antitumor value in MDR BC. The formulated NPs, along with encapsulated doxorubicin, showed sufficient accumulation in tumor cells. The release of the drug was remotely operated by mild near-infrared irradiation. The formulation improved the cell-killing effect by inducing apoptosis. In addition to this, it also prevented the growth of tumor cells by the arrest of the cell cycle and initiated apoptosis by suppressing Src/FAK/AKT signaling pathways. The combination of selenium photothermal-chemotherapy significantly suppressed the growth of the tumor and slowed down its advancement. Visible signs of organ impairment or toxicity were not detected, which was further confirmed by hematology and biochemical analysis. Therefore, the prepared formulation showed the possibility of treating metastatic BC with less or no significant adverse effects [186]. Gold NSs tagged with trastuzumab were administered to trastuzumab-resistant HER2-tumor xenografts. NSs showed a time-dependent increase in accumulation in the tumor tissue after administration of NSs intravenously, followed by irradiation with the femtosecond-pulsed laser. The results revealed significant inhibition of tumor proliferation. This study concluded that photothermal therapy and gold NS-tagged with trastuzumab may be used to overcome trastuzumab resistance [187]. In a similar study, plasmonic NSs made up of polycaprolactone and gold were synthesized, which showed effective/significant localized photo-thermolysis in different cell lines irrespective of receptor makeup. These NSs were capable of producing standalone and considerable eradication of cancer cells without chemotherapeutic agents [188].

### 4.8. Viral Nanoparticles

Nucleic acid/drug loaded into the empty virus capsid once the virulence is removed from its genetic material is referred to as a viral nanoparticle. This transporter system has various advantages gained from the structural arrangement of NPs and naturally responsive capsid surfaces. NPs utilizing nonpathogenic viruses (plant or synthetic) are emerging as an innovative drug delivery strategy as far as nanotechnology is concerned. Plant viruses, when formulated as viral NPs, showed clear advantages over any synthetic ones, the reason being their biocompatible and biodegradable nature. They also have an added benefit of being inexpensive when compared to synthetic nanoparticles. Virus X, extracted from potato, was used as a carrier for chemotherapeutic drug herceptin. Herceptin showed a therapeutic effect against HER2-positive BC cells in cancer patients. Researchers have conjoined potato virus X along with herceptin (trastuzumab), a monoclonal antibody for targeting HER2-positive BC cells. The bioconjugation was carried out in two steps in the presence of EDC/sulfo-N-hydroxysuccinimide (sulfo-NHS) [189]. Conjugation efficiency was examined by various methods such as sodium dodecyl sulfate-polyacrylamide gel electrophoresis (SDS-PAGE), Western blot, and ELISA. These methods confirmed the formation of a protein band formed as a result of conjugation. The bioconjugate could induce apoptosis in SKOV-3 and SK-BR-3 cells (HER2-positive cell lines). The flow cytometry assay measured apoptosis caused by the bioconjugate and compared it with herceptin alone. From the result obtained, it was concluded that potato virus X–herceptin conjugate had enabled herceptin to become a more potential anticancer drug for treating breast cancer [190].

Tobacco mosaic virus (TMV) is a hollow cylindrical nanotube which is converted into spherical nanoparticles of around 50 nm by a heat-transformation method. These spherical TMVs were loaded with doxorubicin, which showed similar antiproliferative effects as that of free doxorubicin on MDA-MB-231 and MCF-7 [191].

## 5. Artificial Intelligence in Personalized BC Therapy

The development of artificial intelligence (AI) in healthcare, especially in the field of cancer, has paved the way for the implementation of personalized medicine. As BC is cancer with complex heterogeneity, personalized medicine based on the genetic and molecular data can be an alternative for its effective treatment. The breakthrough research was put forth by a group of scientists at the Institute of Cancer Research used AI to identify different subtypes of BC where patients responded differently to standard therapy based on the critical differences among the cancer types. Research is currently underway to identify the differences in the genetic and molecular patterns of the BC patients and to include them in clinical trials to implement personalized therapy as a standard treatment. AI was able to identify the cancer subtypes based on the vast amount of data available on the genetic and molecular makeup of cancer, especially luminal cancer, along with patient survival data [192]. The difference in the markers identified in the patients using AI was able to suggest possible treatment implications. This unique advantage of AI in personalizing treatments based on genetic sequences, can be explored for enhanced efficacy in BC patients, including those with MDR BC. AI can also be successfully interfaced with nanomedicine, specifically in the optimization of combination drug therapy based on the stratification of patients as per the BC type and to maintain the drug levels at the target site [193]. In addition, AI may also aid in the selection of materials for the preparation of nanocarriers based on their interaction with drug targets, biological fluids, and cell membranes, which ultimately affect therapeutic efficacy [194]. However, challenges such as data integrity, a huge amount of data and translation of this data into knowledge, ethical consideration, and regulatory approvals lay back the clinical translation of AI [195].

## 6. Toxicity Associated with Advanced Nano-Based Therapy of BC

**Figure 6:**
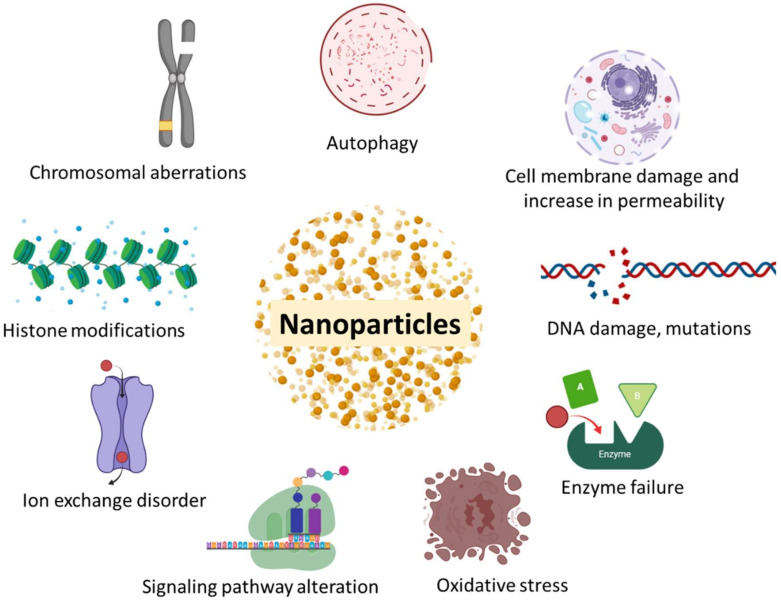
Toxicity associated with nanoparticles.

NPs are available currently, even though considered to be less toxic and more effective drug carriers, they are associated with a certain degree of toxicity (Figure 6). It is reported that after the administration of NPs, there is an increase in the phagocytic cell count and a reduction in the immunological defences of the body. NPs cause cytotoxicity by stimulating the release of pro-inflammatory factors [196]. They also have the ability to accumulate around the protein, depending on their shape, particle size, curvature, and free energy surface charge, which may cause crosslinking and unfolding of proteins, and lead to a loss of enzymatic activity and cell death [197].

Immune checkpoint inhibitors have shown to cause autoimmune responses and may cause psoriasis, uveitis, inflammatory colitis, and maculopapular rash. The adverse effect of a combination of immunotherapy is much more complicated and difficult to handle [198,199,200].

It was observed that after the administration of gene-therapy doses, the immune system of the patient might react and result in a drop in blood pressure, fever, headache, nausea, and vomiting. As gene-therapy is a comparatively newer therapy, the long-term side effects are yet to be established [201].

## 7. Summary and Future Perspective

Breast cancer is a complex system with enormous heterogenicity within itself. Conventional therapy lacks specificity to the tumor tissue, causing intolerable side effects and leading to the development of resistance. Over the years, there has been an improved clinical outcome owing to the latest developments in both immunotherapy and nanotechnology. For HR-positive BC, anti-estrogens, along with aromatase inhibitors, have proven to be a powerful therapy. MAbs against HER2-receptors, with tagging of chemotherapeutic agents (ADC), has shown drastic improvement in the therapeutic outcome. TNBC, being the most aggressive and challenging to treat, can be effectively treated with immune checkpoint inhibitors along with chemotherapeutic agents. PARP inhibitors and drugs targeting the RANK/RANKL system are the latest drugs that have added on to the list of BC drugs. In the past few years, extensive research has been undertaken to overcome resistance to BC therapy and improve its prognosis. NPs loaded with chemotherapeutic agents, along with surface modifications, have been extensively studied and have proven to be an effective treatment strategy with minimal systemic side effects. NPs such as theranostics, which have the combined potential of both diagnosing and treating, can be tailored as a personalized medicine for BC. The major challenge faced by researchers is the identification of suitable biomarkers that are responsible for the development of resistance and response. The development of resistance to current therapy has become a cause of concern, limiting the effectiveness of treatments. One of the breakthrough strategies proven to reverse resistance is the use of multimodal therapy. Nevertheless, the development of resistance and intolerance to current therapy needs to be addressed to improve the overall therapeutic outcome and morbidity in BC patients.

Many new agents and new combination strategies are under clinical investigation, and if successful, could be translated clinically, which will be a ray of hope for treating BC effectively. In addition, BC therapy can be further revolutionized by adopting a personalized treatment strategy based on the biological and molecular specification of individual patients so that the right dose and regimen for treatment can be optimized. Treatment strategies can be designed with the aid of gene profiling to identify genetic biomarkers. However, the major challenge lies in the effective clinical translation of this approach due to the complexities involved in regulatory approvals and data integrity.

## 8. Conclusions

A gradual but continuous decrease in the mortality rate of BC patients has been observed in the past few decades. The prime reason behind this progress is the advancement in the technologies and approaches adopted. Targeted drug delivery systems and multimodal therapy have proven to be more efficacious in treating BC with minimal systemic toxicity. Immunotherapy is well tolerated when compared to chemotherapy and has shown a relatively lower adverse effect. There is a need for a more profound understanding of molecular mechanisms and biomarkers involved in BC development and the mechanism by which cancer cells develop resistance. Stratification of patients, according to specific biomarkers, plays a crucial role in the effective treatment of BC, whereby the symptoms of patients can be ameliorated, thereby improving the quality of life of patients. Treatment strategies/approaches should be tailored as per individuals for effective treatment.

## Figures and Tables

**Table 1:** The potency of cyclin-dependent kinase (CDK)-inhibitors **[15]**.

Agent	Cyclin-Dependent Kinase IC_50_ (in nM)	
	CDK6	CDK4
Ribociclib	39	10
Palbociclib	15	11
Abemaciclib	9.9	2

**Table 2:** CDK4/6 inhibitors with other chemotherapeutic agents in clinical trials as combination therapy.

Drug	Combination With	Description	ClinicalTrials.Gov Identifier NCT Number	Phase
Palbociclib	Endocrine therapy (exemestane, fulvestrant, letrozole, tamoxifen) Chemotherapy (capecitabine, epirubicin, paclitaxel, vinorelbine)	To evaluate/compare palbociclib in combination with endocrine therapeutic agents and with chemotherapeutic agents in HER2-positive or HER2-negative metastatic BC patients	NCT03355157	IV
Palbociclib	Fulvestrant, exemestane, letrozole, anastrozole	To evaluate eHealth-support in locally advanced or metastatic BC patient’s life quality when treated with palbociclib with endocrine therapy	NCT03220178	IV
Ribociclib	Endocrine therapy	To study the effectiveness of ribociclib with endocrine therapy in the ER-positive BC patients	NCT03285412	II
Ribociclib	Letrozole/anastrozole, goserelin	To evaluate the safety and efficacy of 400 mg of ribociclib when given in combination with aromatase inhibitors in the post- and pre-menopausal women with HR-positive/HR-negative patients with advanced BC	NCT03822468	II
Ribociclib	Letrozole 2.5 mg, doxorubicin, cyclophosphamide, paclitaxel	To evaluate the efficacy and safety of ribociclib (LEE011) with multi-agent chemotherapy or letrozole in postmenopausal patients with luminal B/HER2 negative BC	NCT03248427	II
Ribociclib	Non-steroidal aromatase inhibitors- letrozole 2.5 mg/day or anastrozole 1 mg/day orally, LHRH agonist-triptorelin 3.75 mg or leuprolide 3.75 mg or goserelin 3.6 mg, as injectable	Ribociclib in combination with a non-steroidal aromatase inhibitor/LHRH agonist in HR-Positive/HER2-negative patients with advanced BC	NCT03944434	II
Ribociclib	PDR001, fulvestrant	Ribociclib (LEE011), in combination with PDR001 an immunotherapeutic drug and fulvestrant in HR-positive, HER2-negative patient for metastatic hormone-receptor-positive (HR+), HER2-negative BC.	NCT03294694	I
Ribociclib (LEE011)	Paclitaxel	To assess the safety and dose escalation of paclitaxel with ribociclib in retinoblastoma (Rb)-positive patients with advanced BC.	NCT02599363	I
Abemaciclib	Fulvestrant	To compare the effectiveness of combination therapy of abemaciclib with fulvestrant and chemotherapy in HR-positive, HER2-negative patients with metastatic BC (visceral Metastases)	NCT04031885	IV
Abemaciclib	Fulvestrant	To compare fulvestrant alone with a combination of fulvestrant and abemaciclib in progression-free survival in HR-positive and HER2-negative patients.	NCT02107703	III
Abemaciclib	Atezolizumab (MPDL3280A), bevacizumab, entinostat, exemestane, fulvestrant, ipatasertib, tamoxifen	To evaluate the safety, efficacy, and pharmacokinetics of immunotherapy-based combination with CDK 4/6 inhibitor and anti-estrogen agents in advanced or metastatic HR-positive and HER2-negative patients.	NCT03280563	I

**Table 3:** PI3K–AKT–mTOR pathway inhibitors.

Receptor	Drugs	Mechanism of Action
PI3K	Taselisib (GDC0032), pilaralisib (XL147), alpelisib (BYL719), buparlisib (BKM120), pictilisib (GDC0941), MLN1117	PI3K inhibition
AKT	Capivasertib (AZD5363), uprosertib (GSK2141795), miransertib (ARQ092), MK2206	Inhibition of AKT 1,2,3 isoforms
mTORC1	Sirolimus, ridaforolimus, everolimus, temsirolimus	Allosteric inhibition of mTORC1
PI3K and mTOR	Dactolisib (NVP-BEZ235), gedatolisib (PF05212384), LY3023414	Dual inactivation of PI3K and mTOR
mTORC1 and mTORC2	Vistusertib (AZD2014), sapanisertib (TAK-228), CC-223, AZD8055, MLN0138,	Allosteric inhibition of mTORC1 and mTORC2

**Table 4:** List of drugs/combinations of PI3K/AKT/mTOR inhibitors and other agents under clinical trials.

Drug	Combination With	Study Arm	Description	ClinicalTrials.Gov Identifier: NCT Number	Phase
Taselisib	Taselisib, trastuzumab emtansine, pertuzumab, trastuzumab paclitaxel	Arm A: Taselisib with trastuzumab emtansine Arm B: Taselisib with trastuzumab emtansine and pertuzumab Arm C: Taselisib with pertuzumab and trastuzumab Arm D: Taselisib with pertuzumab, trastuzumab, and paclitaxel	Combination of taselisib (GDC-0032) with anti-HER2 therapies in participants with advanced HER2+ breast cancer—a Phase Ib dose-escalation trial	NCT02390427	Ib
Pilaralisib (XL147)	Letrozole (Femara)	Arm A: Pilaralisib with letrozole	A Phase 1/2 dose-escalation study of XL147 (SAR245408), or XL765 (SAR245409) in combination with letrozole performed on subjects with hormone receptor-positive and HER2-negative breast cancer refractory to a nonsteroidal aromatase inhibitor	NCT01082068	Completed
Pilaralisib (XL147)	Trastuzumab, Paclitaxel	Arm A: Pilaralisib with trastuzumab Arm B: Pilaralisib with trastuzumab and paclitaxel	To study the efficacy of a combination of pilaralisib with trastuzumab or paclitaxel and trastuzumab in patients with metastatic -BC	NCT01042925	Completed
Alpelisib (BYL719)	Fulvestrant	Arm A: Alpelisib (300 mg; oral; once daily with fulvestrant (500 mg; IM-injection)	To assess the safety and efficacy in men and postmenopausal women patients with advanced-BC	NCT02437318	III
Alpelisib	Fulvestrant	Arm A: Alpelisib 300 mg oral daily with fulvestrant 500 mg intramuscular on in first cycle 1st day and 15th day, and thereafter every 28-day cycle.	To study the molecular features in HR-positive and HER2-negative postmenopausal women with a PIK3CA mutation	NCT03439046	III
MK2206		Arm A: MK-2206 mg orally once a week	To study the efficacy of MK2206, an AKT inhibitor in patients with advanced BC and having AKT mutation and/or PTEN Loss/PTEN mutation and/or PIK3CA mutation	NCT01277757	Completed
Everolimus,	Letrozole, everolimus, TRC105	Arm A: Letrozole 2.5 mg + everolimus 5 mg + TRC105 15 mg/kg i.v Arm B: Letrozole 2.5 mg + everolimus 10 mg + TRC105 15 mg/kg i.v Arm C: Letrozole 2.5 mg + everolimus 5 mg + 10 mg/kg i.v Arm D: Letrozole 2.5 mg + 5 or 10 mg + TRC105 15 or 10 mg/kg i.v.	To study how well a combination of letrozole, TRC105 everolimus works in patients with stage 2 and 3 BC, and how well it is tolerated	NCT02520063	I/II
Everolimus	Palbociclib, exemestane	Palbociclib, everolimus, exemestane are administered in a cycle of 28 days	To study the efficacy of a combination of palbociclib, everolimus, exemestane in HR-positive and HER2-negative patients with metastatic-BC	NCT02871791	I/II
Temsirolimus	Bevacizumab, cetuximab valproic acid	Arm A: Temsirolimus, bevacizumab, cetuximab Arm B: Temsirolimus, bevacizumab, valproic acid Arm C: temsirolimus, bevacizumab	To study the efficacy of bevacizumab and temsirolimus alone or combination with valproic acid or cetuximab in patients with advanced or metastatic- BC	NCT01552434	I
Gedatolisib (PF05212384)	Trastuzumab biosimilar (herzuma)	Arm A: Herzuma + Gedatolisib	To evaluate the safety and antitumor activity of Herzuma^®^ with gedatolisib in HER-2 positive patients with metastatic-BC	NCT03698383	II
Vistusertib (AZD2014)	Palbociclib, fulvestrant	Arm A: A triple combination of AZD2014 + palbociclib +fulvestrant	To study the efficacy of a combination of AZD2014, palbociclib, fulvestrant, in ER-positive patients with metastatic-BC	NCT02599714	I

**Table 5:** List of drugs under clinical trials for the treatment of TNBC.

Drug	Combination With	Study Arm	Description	ClinicalTrials.Gov Identifier: NCT Number	Phase
Cisplatin	Romidepsin, nivolumab	Arm A: cisplatin (75 mg/m^2^) + romidepsin (8 mg/m^2^) Arm B: romidepsin (10 mg/m^2^) + cisplatin (75 mg/m^2^) Arm C: romidepsin (12 mg/m^2^) + cisplatin (75 mg/m^2^) Arm D: romidepsin MTD (maximum tolerable dose) + cisplatin (75 mg/m^2^) + nivolumab 360 mg	To study the efficacy of cisplatin in combination with romidepsin and nivolumab in patients with TNBC or BRCA mutation or metastatic-BC	NCT02393794	I/II
MM-310		Arm A: MM-310 i.v., 21-day cycle.	MM-310 is a docetaxel loaded liposomal formulation with targeting ability of EphA2 receptor. In this study, safety and efficacy of MM-310 in patients with TNBC was studied	NCT03076372	I
Carboplatin	Everolimus	Arm A: Carboplatin alone Arm B: Carboplatin + everolimus	To study the safety and efficacy of carboplatin alone and carboplatin in combination with everolimus in patients with advanced TNBC	NCT02531932	II
Leronlimab	Carboplatin	Arm A: leronlimab (350 mg) + AUC 5 carboplatin Arm B: leronlimab (525 mg) + AUC 5 carboplatin Arm C: leronlimab (700 mg) + AUC 5 carboplatin Arm D: leronlimab (MTD) + AUC 5 carboplatin	To study the safety and efficacy of leronlimab when given in combination carboplatin in the patients with advanced TNBC	NCT03838367	Ib/II
Nab-Paclitaxel	Mifepristone	Arm A: nab-paclitaxel 100 mg + mifepristone 300 mg Arm B: nab-paclitaxel 100 mg + placebo	To study the efficacy of Nab-paclitaxel alone and in combination with mifepristone in the glucocorticoid receptor-positive and TNBC patients	NCT02788981	II
L-NMMA	Docetaxel amlodipine pegfilgrastim Enteric-coated aspirin	Arm A: L-NMMA + docetaxel + amlodipine + pegfilgrastim + Enteric-coated aspirin	To study the L-NMMA’s MTD, dose-limiting toxicities and to find out the efficacy in combination with docetaxel in patients with advanced TNBC	NCT02834403	Ib/II
Mirvetuximab soravtansine	Gemcitabine hydrochloride	Arm A: mirvetuximab soravtansine + gemcitabine hydrochloride	To study the dose-escalation, tolerability safety and efficacy of mirvetuximab soravtansine in combination with gemcitabine hydrochloride in patients with folate receptor-positive ovarian and TNBC	NCT02996825	I
Atezolizumab	Paclitaxel	Arm A: atezolizumab (840 mg) and paclitaxel (90 mg/m^2^) Arm B: placebo and paclitaxel (90 mg/m^2^)	To study the efficacy of a combination of a PD-L1-antibody, atezolizumab, and paclitaxel in advanced TNBC patients	NCT03125902	III
Tak-228	Tak-117 Cisplatin Nab-Paclitaxel	Arm A: Tak-228 and Tak-117 followed by cisplatin and nab-paclitaxel	To study the efficacy of TAK- 228 and TAK- 117 treatment followed by cisplatin and nab-paclitaxel treatment in patients with metastatic TNBC	NCT03193853	II
Onalespib	paclitaxel	Arm A: onalespib (i.v.) on day 7 + paclitaxel (i.v.) days 1, 8, and 15; 28-day cycle	To study the best dose and side effects of onalespib when given in combination with paclitaxel in advanced TNBC patients	NCT02474173	I

**Table 6:** List of approved nanotechnology-based cancer drug therapies.

Product	Drug	Company	Nanoparticle	Composition	Particle Size	Indication	Approval by US FDA and others	Reference
Genexol-PM	Paclitaxel	Samyang/Biopharm	PEG-PLA polymeric micelle	Paclitaxel, monomethoxy poly (ethylene glycol)-block-poly (D,L- lactide)	23.91 nm	Breast, lung, ovarian cancer	2007 (South Korea)	[114,115,116]
Abraxane	Paclitaxel	Abraxis/Celgene	Nanoparticle-bound albumin	Paclitaxel, human albumin	130 nm	In breast, pancreatic and non-small cell lung cancer	2005	[117,118]
Doxil	Doxorubicin	Johnson and Johnson	Liposome	N-(carbonyl-methoxypolyethylene glycol 2000)-1,2-distearoyl-sn-glycero-3-phosphoethanolamine sodium salt (DSPE-PEG 2000), hydrogenated soy phosphatidylcholine, and cholesterol	85 nm	Kaposi’s sarcoma Ovarian cancer Breast cancer Multiple myeloma	1995 1999 2003 2007 (Europe, Canada)	[119,120]
Myocet	Doxorubicin	Cephalon	Liposome	Egg phosphatidylcholine and cholesterol with Doxorubicin citric acid aqueous core	190 nm	Breast cancer	2000 (EU)	[119,120]
Depocyt	Cytarabine	Pacira	Liposome	Cholesterol, glycerol trioleate, triglyceride, phospholipids (dipalmitoyl phosphatidylglycerol), and dioleoyl phosphatidylcholine	20 µm	Neoplastic meningitis	1999	[121,122,123]
Lipo-Dox	Doxorubicin	Taiwan Liposome	Liposome	Cholesterol, N-(carbonyl-ethoxypolyethylene glycol 2000)-1,2-distearoyl-sn-glycero-3-phosphoethanolamine, hydrogenated soybean lecithin	104.2 nm	Breast, ovarian cancer	1998 (Taiwan)	[124,125]
DaunoXome	Daunorubicin	Galen	Liposome	Cholesterol, distearoylphosphatidylcholine	45 nm	Kaposi’s sarcoma	1996	[126,127]

**Table 7:** List of some nanomedicines explored for the treatment of breast cancer.

Nanoparticle Type	Type of Breast Cancer	Therapeutic Agent	Materials Used	Entrapment Efficiency (EE) and Particle Size	Key Outcome	Reference
Liposomes	luminal breast cancer	Anti-IL6R antibody, Doxorubicin	Cholesterol, 1,2-dioleoyl-sn-glycero-3-phosphoethanolamine 1,2-dioleoyl-sn-glycero-3-phosphocholine In (1:1:1 molar ratio)	85.81% ± 0.4799% to 89.03% ± 0.143%, ∼100 nm	Formulation showed an enhanced tumor targeting efficacy with anti-tumor metastasis effects in BALB/c mice bearing 4T1 cells	[128]
Liposomes	Luminal breast cancer- Estrogen positive BC	Doxorubicin	Estrone conjugated DPPC and DSPE-PEG2000-NH_2_ liposomes	EE not reported, 194 nm	Formulations showed significant uptake in ER-positive (MCF-7) and non- significant uptake in ER-negative (MDA-MB-231) cell lines	[129]
Albumin nanoparticles	HER2 positive BC	2-methoxy-estradiol	Bovine serum albumin	89.85% ± 3.80% to 88.70% ± 2.95%, ∼238.8 ± 5.1 nm	Formulated NPs showed enhanced cytotoxicity and cellular uptake when compared with the free drug when assessed in SK-BR-3 and MCF-7 cell line and SK-BR-3, MCF-7 tumor-bearing mice	[130]
chitosan nanoparticles	HER2 positive BC	doxorubicin	O-succinyl chitosan graft Pluronic^®^ F127 and 5% to 10% anti-HER2 peptide	73.69% ± 0.53% to 74.65% ± 0.44%, 34.92 to 50.79 nm	In vitro cytotoxicity and cellular-uptake study were performed on MCF-7 cell line which showed that the NP conjugated with anti-HER2 showed higher cytotoxicity when compared with free drug	[131]
liposomal	HER2 positive BC	doxorubicin	HER2pep-K3-palmitic acid conjugate, DSPC, mPEG2000-DSPE	>98%, ~80 nm	In vitro cytotoxicity and cellular-uptake studies were performed on BT-474, SK-BR-3 and MCF-7. The formulation showed higher cytotoxicity and cellular uptake with lower systemic toxicity when compared with free drug	[132]
Iron oxide	HER2 positive BC	siRNA	Iron oxide, caffeic acid, calcium phosphate and PEG-polyanion block copolymer	EE not reported, 130 nm	In vitro cytotoxicity and cellular-uptake studies were performed on HCC1954. After treating with NP, HER2 mRNA expression was decreased by 38% when compared with naked siRNA	[133]
Polymeric nanoparticles	HER2 positive BC	Emtansine	D-α-tocopheryl polyethylene glycol 1000 succinate-poly (D, L-lactide)	84–94%, 102–125 nm	In vitro cytotoxicity and cellular-uptake studies were performed on MDA-MB-453 cell lines and in vivo cytotoxicity study in MDA-MB-453 xenograft mice model. The nanoparticle showed superior antitumor effect when compared with the free drug	[134]
Polymeric nanoparticles	TNBC	Paclitaxel	Poly(lactic-co-glycolic acid) NP coated with hyaluronic acid	84–98.34%, 225.1±0.43 nm	In vitro cell viability and cellular-uptake studies were performed on MDA-MB-231. The prepared NP showed an improved cellular uptake and thereby higher cytotoxicity in cancer cells when compared with free drug	[135]
SLN	TNBC	Di-allyl-disulfide	SLN prepared with palmitic acid, soya lecithin and pluronic F-68 and surface modified with glycation end products antibody	79.23%, 116.20 nm	Formulated NP demonstrated a high cellular uptake by MDA-MB231 cell line and thereby reduction in systemic side effect of the drug and increased activity at tumor site	[136]
Polymer nanoparticles	TNBC	Curcumin,	Chitosan NPs with apoptosis-inducing ligand (TRAIL)	51.67%, 652 ± 10 nm,	The NPs showed a reduced tumor volume when compared to control when tested in BALB/c mice	[137]
Iron oxide NPs	TNBC	Baicalein	PEG-coated iron oxide magnetic nanoparticles	95.3%, 100 nm	Significantly inhibited the MDA-MB-231 cell growth when tested in vitro and showed significant anti-apoptotic activity	[138]
Polymeric NPs	TNBC	Thymoquinone	Hyaluronic acid conjugated Pluronic^®^ P123 and Pluronic^®^ F127 NPs	EE not reported, 22.0 ± 3.1 nm	Formulations retarded cell growth and migration of MDA-MB-231. Studies in Balb/c mice showed significant reduction in tumor load when treated with formulations	[139]

**Table 8:** List of liposome-based drug products approved by the US FDA and under clinical investigation.

Product	Drug	Manufacturer	Indications	US FDA Approved Date/Clinical Trial Status
Doxil (Caelyx)	Pegylated doxorubicin	Orthobiotech, Schering-Plough	Ovarian/breast cancer	November 1995
Myocet	Liposome-encapsulated Doxorubicin	Elan/Sopherion Therapeutics	Breast cancer	2000, Approved in Europe and Canada
LEP-ETU	Liposomal Paclitaxel	Neopharma	Ovarian/breast/lung cancers	Phase I/II
EndoTAG-I	Paclitaxel	Medigene/SynCore Biotechnology	Breast cancer/pancreatic cancer	Phase II
Genexal-PM	Paclitaxel-loaded polymeric micelle	Samyang	Breast cancer/small cell lung cancer	Marketed in Europe, Korea
Nektar -102	Irinotecan, PEGylated liposome	Nektar therapeutics	Breast/colorectal cancer	Phase III
